# Tissue Factor, Blood Coagulation, and Beyond: An Overview

**DOI:** 10.4061/2011/367284

**Published:** 2011-09-20

**Authors:** Arthur J. Chu

**Affiliations:** Division of Biological and Physical Sciences, Delta State University, Cleveland, MS 38733, USA

## Abstract

Emerging evidence shows a broad spectrum of biological functions of tissue factor (TF). TF classical role in initiating the extrinsic blood coagulation and its direct thrombotic action in close relation to cardiovascular risks have long been established. TF overexpression/hypercoagulability often observed in many clinical conditions certainly expands its role in proinflammation, diabetes, obesity, cardiovascular diseases, angiogenesis, tumor metastasis, wound repairs, embryonic development, cell adhesion/migration, innate immunity, infection, pregnancy loss, and many others. This paper broadly covers seminal observations to discuss TF pathogenic roles in relation to diverse disease development or manifestation. Biochemically, extracellular TF signaling interfaced through protease-activated receptors (PARs) elicits cellular activation and inflammatory responses. TF diverse biological roles are associated with either coagulation-dependent or noncoagulation-mediated actions. Apparently, TF hypercoagulability refuels a coagulation-inflammation-thrombosis circuit in “autocrine” or “paracrine” fashions, which triggers a wide spectrum of pathophysiology. Accordingly, TF suppression, anticoagulation, PAR blockade, or general anti-inflammation offers an array of therapeutical benefits for easing diverse pathological conditions.

## 1. Introduction: Tissue Factor Biology

Tissue factor (TF), also known as factor III, essentially provides additional protection to vital organs prone to mechanical injury; its strategic location is considered as a hemostatic envelope for arresting bleeding from vascular beds. High TF expression is found in highly vascularized organs (cells) such as the brain (e.g., astrocytes), placenta (e.g., trophoblasts), and the lungs (e.g., alveolar cells) followed by the heart (e.g., cardiac myocytes, pericytes, fibroblasts), kidney, intestine, testes, and uterus (e.g., epithelial cells surrounding the organs). The low expression is detected in the spleen, thymus, and liver [1]. Circulating (blood-borne) TF is mainly derived from its expression in blood cells (e.g., monocytes, macrophages, granulocytes, and platelets), platelet-free microparticles containing TF shed from cells, or even soluble TF protein; the serum level can be easily measured by TF antigen, ELISA, TF procoagulant activity (PCA), and so forth (for review, see [2]).

**Figure 1:**
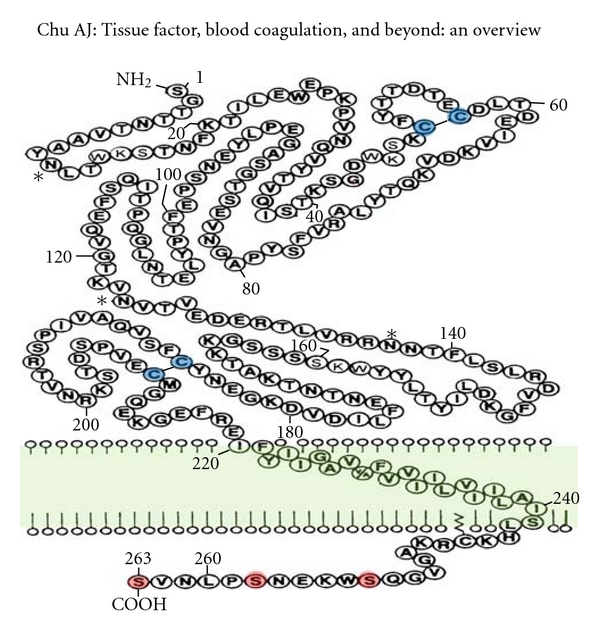
TF structure. The 46-kDa membrane-bound single polypeptide chain consists of extracellular, transmembrane, and cytoplasmic domains. There are 2 intrachain disulfide bridges (Cys49-Cys57 and Cys186-Cys209, shown in blue) in the extracellular domain where it also contains FVII/FVIIa binding domain (see text) initiating signaling cascade for the extrinsic blood coagulation (Figure 2, left panel). There are three serine residues (shown in red) in the cytoplasmic domain for undergoing phosphorylation. *Denotes 3 proposed glycosylation sites at Asn11, 124, and 137. Adopted and modified from [3].

Full-length TF (Figure 1), a membrane integral glycoprotein (46 kDa), is a 263-amino acid single-chain polypeptide classified as CD142 (Type II cytokine receptor) with a 219-amino-acid extracellular *N*-terminus and a 23-amino-acid transmembrane domain followed by an intracellular 21-amino-acid *C*-terminus [3, 4]. The extracellular region contains FVII/VIIa binding domains. Extracellular soluble form (sTF) could be released from EC [5] in response to proinflammatory cytokines. The intracellular domain could undergo serine phosphorylation(s), which could modify its function [6–8]; for instance, the cytoplasmic domain negatively regulates TF expression, which is mediated by suppressed Erk1/2 phosphorylation. 

TF initiates the extrinsic coagulation that plays an integral role in blood coagulation, thrombin (FIIa) generation, and thrombi formation in close relation to thrombosis and cardiovascular dysfunctions [9, 10]. Such extracellular TF signaling proceeds with the sequential generation of coagulant mediators (FVIIa, FXa, and FIIa: active serine proteases) and fibrin production, all of which are proinflammatory [4]. TF extends its roles to diverse biological phenomena related to either ro both of these two major thrombotic and inflammatory events. Emerging evidence shows TF involvement in wound repairs, embryonic development, angiogenesis, tumor metastasis, cell adhesion/migration, innate immunity, and many pathological conditions. 

## 2. Regulation of Tissue Factor Expression

TF usually in its latent (cryptic) form is often upregulated (decrypted) upon vascular injury (by protein disulfide isomerase with phosphatidylserine (PS) exposure) [10–12], inflammation (e.g., LPS, ILs, TNF-*α*, C-reactive protein (CRP),* C. pneumoniae*), IFN, MCP-1, ICAM, *P*-selectin, CD40/40L, PDGF, OxLDL, Lp(a), angiotensin II, plasmin, complement anaphylatoxin C5a, antiphospholipid antibody (aPL), advanced glycation endproducts (AGEs), hypoxia, and so forth (for review, see [4]) in cultures. Enhanced TF expression has also been reported due to SirT1 inhibition [13], homocysteine [14], oral contraceptives [15], shear stress [16], amyloid protein A [17], histamine [18], smoking [19], nicotine [20], estrogen [21], asbestos [22], serotonin [23], dexamethasone [24], arachidonic acid (AA) [25], bFGF [26], VEGF [27], EGF [28], aggregated LDL [29], leptin [30], urokinase [31], shingosine-1-phosphate [32], or many others. In general, TF expression is mediated by activations of intracellular signaling kinases (e.g., PKC, MAPK (Erk, p38)) and other signaling components such as transcription factors (e.g., AP-1, NF*κ*B, Erg-1) (for review, see [4]). Exposure to calcium ionophores such as A23187 drastically sustains cellular TF PCA without increased TF expression in cultures, which could either have or not have any pathological implications, and the mechanism of action remains unclear [4]. 

In contrast, its downregulation has been reported including by HMG-CoA reductase inhibitors [33, 34], cyclooxygenase (COX) inhibitors [35–37], paclitaxel [38], lysophosphatidylcholine [39], insulin [40], nicotinamide [41], nitric oxide (NO)/or soluble guanylate cyclase activator [42], hydroxyurea [43], ethyl pyruvate [44], dimethyl sulfoxide (DMSO) [45], angiotensin converting enzyme (ACE) inhibitors [46], adiponectin [47], retinoic acid [48], all-trans retinoic acid [49], vitamin D3 [50], PGJ2 [51], PPAR*α* agonists (fenofibric acid, WY14643, and GW2331) [52]/activators (WY14643 and eicosatetraenoic acid) [53], liver X receptor agonists [54], pentroxifylline [55], phenolics/resveratrol derivatives [56], indobufen [57], amiodarone [58], metformin [59], elevated intracellular cAMP [4], and PI3K/Akt/PKB signaling [60]. On the molecular biology front, miR-19 [61], short hairpin RNA [62], hairpin ribozyme [63], or antisense ODN [64–66] readily downregulates TF mRNA translation and expression.

## 3. TF-Initiated Extrinsic Coagulation

**Figure 2:**
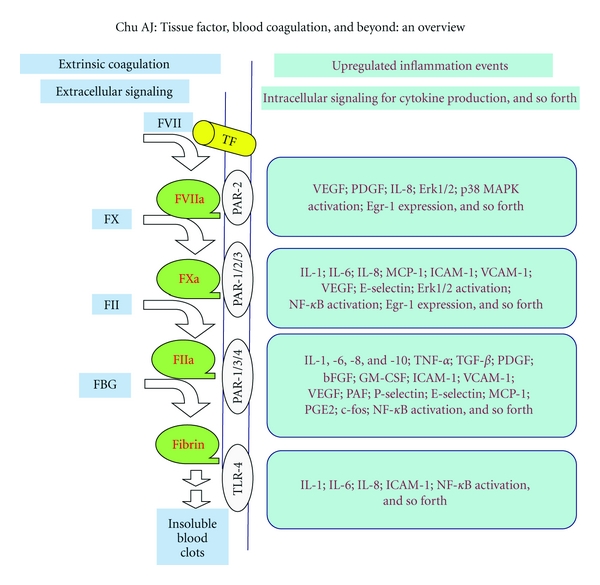
TF hypercoagulability and inflammation. TF-initiated extrinsic coagulation (left panel) essentially proceeds as extracellular signaling and results in the generation of active serine protease (coagulant mediators: FVIIa, FXa, and FIIa) derived from their corresponding zymogen activations. FBG is cleaved by FIIa to produce fibrin that is polymerized and cross-linked to yield insoluble blood clots. Such TF extracellular signaling activates cells for proinflammation. Through cell receptors on plasma membrane, signals from the coagulant mediators (FVIIa, FXa, and FIIa) as well as fibrin mediate diverse intracellular activation and the production of proinflammatory mediators (right panel) including cytokines, adhesion molecules, and growth factors, PAR: protease activated receptor; TLR: Toll-like receptor; IL: interleukin; NF*κ*B: nuclear factor kappa B.

In a classical view, TF initiates the extrinsic blood coagulation, which proceeds as Ca+2-dependent extracellular signaling to sequentially activate zymogens: FVII, factor X (FX), and prothrombin (FII) for the generation of coagulant mediators (active serine proteases): FVIIa, FXa, and thrombin (FIIa), respectively. As a result, FIIa cleaves off fibrinogen (FBG) into fibrin monomers that cross-link to produce insoluble blood clots. The extrinsic pathway plays an integral role in blood coagulation complemented by the intrinsic pathway that ensures FIIa regeneration and clot production (Figure 2, left panel) (for review, see [3, 4, 10, 67]). The intrinsic pathway merging with TF-initiated extrinsic coagulation at FX activation is beyond the focus of this paper.

### 3.1. FVII Activation

FVII readily undergoes proteolytic activation of peptide bond cleavage between Arg 152 and Ilu 153 by either TF dependence or other serine proteases (e.g., FXa, FIXa, FXIa, FXIIa, FIIa, or plasmin), resulting in two smaller chains of FVIIa. The *N*-terminus-derived light chain (~20 KDa) contains the membrane-binding Gla domain, while the *C*-terminus-derived heavy chain (~30 KDa) contains the catalytic domain. 

### 3.2. TF-Dependent FVII Activation

The ability of FVII to bind its cofactor (TF) has been reported with a-1 : 2 stoichiometric ratio. It has long been established that Ca+2 and membrane anionic phospholipids are required for TF-dependent FVII activation. Gla, EGF-1, EGF-2, and protease domain (PD) in FVII make essential contributions to the optimal interaction/binding with its counterpart: extracellular sTF1-219. It is said that zymogen FVII affinity for sTF causes secondary conformational changes of the PD, dictating the protease activity. EPR study shows multiple contacts between two proteins; the Gla binds sTF158 and 207, the EGF-1 binds sTF22 and 140, and the PD binds sTF45 and 94. In contrast, Gla-domainless-FVII shows a rapid loss in FVII binding affinity for TF. FVII affinity is also altered upon modification/conformational changes involving the EGF-1 region. Accordingly, any FVII global conformational misfolding/unfolding disrupting the binding sites could result in impaired FVII activation.

Furthermore, there are high-affinity Ca+2 binding sites in Gla, EGF-1, and PD. It has been reported that one Ca+2 molecule binds to PD, another Ca+2 binds to the EGF-1 domain at a high-affinity site, and seven more Ca+2 molecules bind with variable affinity for the Gla domain. Thus, it seems likely that Ca+2 could play a critical role in FVII binding to TF. In addition to PS being essential for TF-dependent FVII activation, cholesterol enrichment of primary human monocyte-derived macrophages also drastically increases TF PCA [68].

### 3.3. Downstream Sequential Activations

The catalytic function of the binary complex TF/FVIIa relying in mutual binding conformation is believed to be directly responsible for FX and FIX activation. FXa acts as a molecular switch not only receiving the upstream (extrinsic and intrinsic) signals but also dictating the downstream coagulation. Strategically, FXa is an active enzyme component coupled with FVa in prothrombinase complex located at the center of the blood coagulation cascade, which converges the clotting signals derived from both the extrinsic (FVII activation) and intrinsic (FIX activation) pathways. FXa also undertakes a feedback activation of FVII. Finally, FIIa derived from FII cleavage by FXa assumes the main coagulant function at the termination stage; it directly catalyzes FBG cleavage releasing fibrinopeptides for fibrin clot production upon cross-linking. In addition, FIIa activates FXIII, FXI, FVIII, or FV, assuring the propagation of blood coagulation. 

## 4. TF Hypercoagulability Leading to Thrombosis

**Figure 3:**
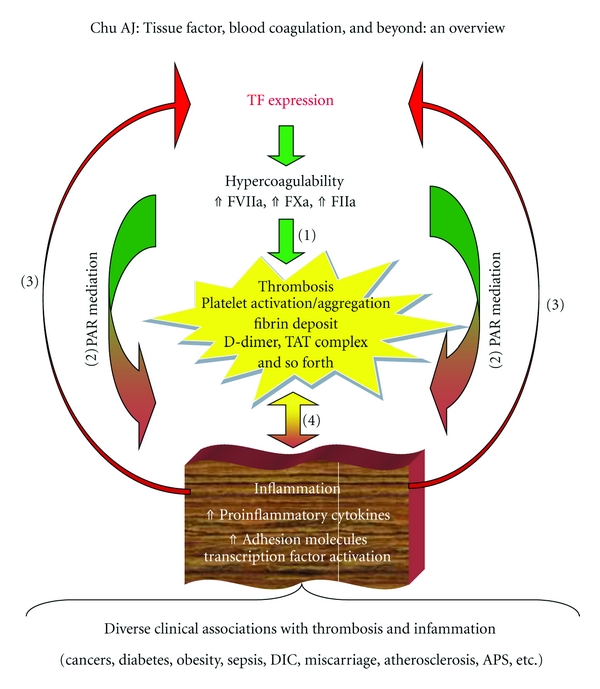
Coagulation-inflammation-thrombosis circuit. TF hypercoagulability results in direct thrombotic actions (**1**). TF also plays converging and diverging roles in driving the coagulation-inflammation cycle ((2) coagulation-dependent inflammation and (3) inflammation-dependent coagulation). Namely, TF hypercoagulability could result in enormous inflammation as the result of continuously refueling the cycle in which coagulation and inflammation promote each other upon the cycle gaining its initial momentum. Thrombosis-inflammation connection (4) is incorporated into the coagulation-inflammation cycle to form a complete coagulation-inflammation-thrombosis circuit, which manifests diverse pathological conditions in relation to inflammation and thrombosis, including cancers, APS, cardiovascular dysfunctions, diabetes, obesity, and DIC.

As a consequence of TF hypercoagulation, thrombosis featuring fibrin overproduction is a direct outcome (Figure 3(1)) in addition to proinflammatory environment for thrombogenesis (i.e., inflammation-dependent thrombosis discussed in Section 7.1). Moreover, elevated FIIa generation upon hypercoagulation impacts thrombogenesis by severalfold relevance to platelet activation/aggregation, clot stabilization, and antifibrinolysis (for review, see [69, 70]).

FIIa activates platelets mainly through protease-activated receptor (PAR) and glycoproteins (GPs) IIb/IIIa, and GPIb. PAR-1 is a primary receptor for FIIa by which platelets are activated to aggregate [71]. Platelet aggregation constitutes thrombus formation involving cross-linking of adjacent platelets mediated by the interaction of activated GP IIb/IIIa with distinct amino acid sequences, LGGAKQAGDV, and/or RGD, at each end of dimeric FBG molecules [72]. Alternatively, FIIa-induced platelet activation could result from polymerizing fibrin, which involves the recognition sites in the cross-linking of polymerizing fibrin and surface integrins via GP Ib. In fact, GP Ib acts as an FIIa-binding site and promotes platelet activation by low FIIa concentrations [73]. In addition, FIIa activates FXIII, and FXIIIa facilitates the stabilization and cross-linking of fibrin clots. 

Concerning hemostatic imbalance with suppressed fibrinolysis, FIIa activates plasma carboxypeptidases recognized as thrombin activatable fibrinolytic inhibitor (TAFI) that attenuates fibrinolysis [74] thereby in favor of fibrin deposition/accumulation. Subsequently, TAFI inhibits various forms of plasminogen activator- (PA-) mediated fibrinolysis [75]. Upregulated plasminogen activator inhibitor-1 (PAI-1) expression by FIIa via a PKC-dependent mechanism [76] could further contribute to antifibrinolytic process and fibrin accumulation.

## 5. Coagulation-Dependent Inflammation

Several lines of evidence reveal* in vivo *coagulation-dependent inflammation. PARs generally mediate inflammation derived from coagulant mediators (e.g., FVIIa, FXa, and FIIa) and fibrin (Figure 2; right panel). Moreover, deficiencies in natural anticoagulants (e.g., tissue factor pathway inhibitor (TFPI), antithrombin (AT III), and activated protein C (APC)) are often susceptible to sepsis [77], disseminated intravascular coagulation (DIC) consequences [78], and inflammation [79]. Consistent with such notion of coagulation-dependent inflammation, anticoagulation readily results in anti-inflammatory effects *in vivo* and *in vitro *(discussed in Section 10). 

### 5.1. Coagulant Mediators Are Proinflammatory

#### 5.1.1. TF in Inflammation

sTF1-219 induces inflammatory arthritis [80], which is characterized by elevated plasma IL-6 and paw swelling accompanied by fibrin overproduction and platelet aggregation. TF mediates IL-1*β*-induced vascular permeability, an inflammatory index [81]. Conversely, TF deficiency reduces inflammation [82]. The ability of anti-TF Ab to prevent septic shock [83] and depress macrophage expression of adhesion molecule CD18 [84] is consistent with the proinflammatory function of TF. 

#### 5.1.2. FVIIa in Inflammation

Elevated plasma level of FVIIa shows significant correlations to CRP and IL-6 expression [85], while FVII deficiency protects from acute inflammation [86]. Administration with recombinant FVIIa enhances IL-6 and -8 productions in healthy human subjects [87]. 

#### 5.1.3. FXa in Inflammation

FXa/PL infusion increases IL-6 and CRP in baboons [88]. FXa induces IL-6 [89], IL-8, MCP-1, ICAM/VCAM, and E-selectin expressions [90]. Consistent with the notion of proinflammatory FXa, ZK-807834, an FXa inhibitor, blocks IL-6 elicitation [89]. 

#### 5.1.4. FIIa in Inflammation

FIIa with fibrin (ogen) dependency induces macrophage adhesion and the production of IL-6 and MCP-1 [91]. FIIa signaling elicits IL-6 [92], IL-8 [93], MCP-1 [93], VEGF [94, 95], and ICAM/VCAM expression [96]. FIIa activates platelets releasing proinflammatory serotonin, histamine, and eicosanoid precursors as well as adhesion molecules [97]. 

#### 5.1.5. Fibrin in Inflammation

Fibrin clot per se is proinflammatory. Fibrin enhances not only IL-1*β* production [98], but also NF-*κ*B activation (a hallmark of inflammation) to induce the expression of ICAM-1 and IL-8 [99], which has been proposed to be mediated by Toll-like receptor-4. D-dimers elicit the synthesis [100] and release [100, 101] of IL-1*β* and IL-6, while fragment D or E [101] stimulates IL-1*β* secretion. FBG degradation product D elevates IL-1 to upregulate IL-6 production [102]. Fibrin fragment E enhances IL-6 production [103]. 

### 5.2. Protease-Activated Receptor (PAR) Mediates Inflammation

PARs functioning as molecular switches dictate cross-talks of hypercoagulable states with inflammatory outcomes (Figure 2). PAR expressed ubiquitously in different cell types belongs to the superfamily of GPCR; there are four major isoforms of which the expression is not affected by exogenous LPS, TNF-*α*, IL-1*β*, or IFN-*γ* . PAR activation by their corresponding activating peptides triggers inflammation [4, 104–106]. For instance, PAR-1 [107]/-2 [107–109]/-4 [107] activations lead to enhanced production of IL-6/8 and IL-1*β* [110]. PAR-2 agonists induce TNF*α* [111] and IL-8 [112] secretion, while PAR-1 deficiency reduces inflammation [82]. 

The receptor activation involves a proteolytic cleavage of the extracellular domain, resulting in the formation of a new *N* terminus that in turn acts as a tethered ligand to interact with exoloop 2 Glu^260^ and then activate heterotrimeric G proteins, triggering an array of intracellular signaling cascade. For instance, the involved sequences of PAR-1 (TLDP*R**^41^**S**^42^***FLLRNP) and PAR-2 (SSKG*R**^36^**S**^37^***LIGKY) are cleaved between R and S by serine proteases such as FIIa that also cleaves PAR-3 (TLPI*KT*FRGAP) and PAR-4 (LPAP*RG*YPGQV) at *K/T* and *R/G*, respectively [113].

The ability of PAR per se to mediate inflammatory responses [4, 104–106] is readily in line with coagulation-dependent inflammation. It is now clear that PARs transmit clotting signals for proinflammation (Figures 2 and 3(2)). PAR-1, 3, or 4 is responsible for FIIa signaling. PAR-2 or 3 mediates FXa signaling, while PAR-2 enables FVIIa signaling (Figure 2, right panel). For instance, PAR-1 transmits FIIa signal enhancing the expression of IL-6, -8, TNF*α*, MCP-1, ICAM-1, PDGF (AB/BB), bFGF, TGF*β*, VEGF, *P*-selectin, and Erk/NF*κ*B/iNOS activation, enhanced PI hydrolysis, COX-2 expression, upregulated [Ca+2]i movement, platelet aggregation, and macrophage adhesion.Via PAR-2 signaling, FVIIa activates MAPK and promotes [Ca+2]i movement, while FXa upregulates the expression of IL-6, -8, MCP-1, PDGF, VEGF, as well as NF*κ*B and MAPK activation. PAR-3 mediates FXa and FIIa proinflammation for enhanced cytokine production of IL-6/-8 and adhesion molecule (MCP-1), while PAR-4 transmits FIIa signaling in leukocyte rolling and adhesion (for review, see [4]).

Taken together, it is evident that coagulant mediator (e.g., FVIIa, FXa, and FIIa) generation and fibrin production in the extracellular compartment via PARs signal transduction and intracellular activations result in the productions of cytokines, adhesion molecules, growth factors, and other proinflammatory components. 

## 6. Inflammation Ensuring TF Hypercoagulation:Vicious Coagulation-Inflammation Cycle

In addition to the above-mentioned TF divergent role in coagulation-dependent inflammation, TF converges various inflammatory signals in either local or systemic inflammation; not only “extrinsic”, but also the resulting “intrinsic” ones reversely turn on and activate coagulation (Figure 3(3)). For instance, rIL-6/8 upregulate procoagulation [114]. The intramuscular injection of IL-6 results in FIIa generation in baboons [115]. TNF-*α* upregulates TF expression in ARDS [116]. CRP drastically activates TF expression [117]. Viral infection such as CpG ODN induces TF expression mediated by TLR-9/MyD88-Erk1/2 pathway with Egr-1 activation [118]. Long pentraxin-3, an acute inflammatory molecule, upregulates TF expression in lung injury [119]. Conversely, guggulsterone (an anti-inflammatory phytosterol) inhibits TF expression and arterial thrombosis [120], which is also in favor of such inflammation-triggered coagulation (Figure 3(3)). 

Importantly, mounting evidence supports the existence of such a positive feedback/reversible loop (Figure 3(3)) in a complete coagulation-inflammation cycle [9]. For instance, FVIIa [121], FXa [121–124], FIIa [17, 31, 124], and PAR-1 [125] promote TF expression. PAR-2 agonists (e.g., trypsin [126], SLIGKV [126], and proteinase-3 [127]) induce TF mRNA. Conversely, TF expression is diminished by anticoagulants (e.g., TFPI [128–130], FVIIai [131], DX9065a [120], ZK 807834 [89], low-molecular-weight heparins (LMWHs) [132], heparin [133, 134], hirudin [135, 136], hirulog [137], AT III [138], APC [139]), which is consistent with the positive feedback loop of inflammation-dependent coagulation (Figure 3(2) and (3)) in completing the vicious cycle. In addition, Wakefield and his associates have demonstrated that selectin-deficient mice lacking the activation of the extrinsic pathway are defective in fibrin production [140].

Thus, it is clear that TF initiates cross-talks of hypercoagulable states with inflammatory outcomes (Figure 2). Furthermore, TF hypercoagulability results in enormous inflammation as the result of continuously refueling the coagulation-inflammation cycle ((Figure 3(2)  and (3)) upon gaining its initial momentum such as local or systemic inflammatory/infectious conditions.

## 7. The Paradigm: Coagulation-Inflammation-Thrombosis Circuit

TF hypercoagulability drives “autocrine” and “paracrine” signaling, thereby amplifying, refueling, and ensuring the paradigm: coagulation-inflammation-thrombosis circuit where it includes the direct thrombotic actions (Figure 3(1)), coagulation-dependent inflammation (Figure 3(2)), a positive feedback loop of inflammation-triggered TF expression (Figure 3(3)), and thrombosis-inflammation connection (Figure 3(4)).

### 7.1. Thrombosis-Inflammation Connection (Figure 3(4)) 

Thrombosis and inflammation are two major consequences of blood coagulation, both of which cross-talk and promote each other. Clinical association of thrombosis with inflammation has been reported in many cases [141]. Such inflammation-thrombosis connection (Figure 3(4)) provides an alternative pathway that blood coagulation via its inflammatory consequence indirectly contributes to thrombosis. 

Several lines of evidence reveal thrombosis-dependent inflammation based on the ability of fibrin and its fragments to elicit IL-1*β*, IL-6, and IL-8 expression [98–103]. Further, platelet activation/aggregation participates in complement activation resulting in inflammatory responses. *P*-selectin as a C3b-binding protein sufficiently leads to C3a generation and C5b-C9 formation, which supports a novel mechanism of local inflammation in vascular injury sites [69, 141].

Conversely, *in vivo* inflammation-dependent thrombogenesis also exists. IL-8 enhances fibrosis in rats [142]. In support of this notion, activation and antagonism of proinflammatory PARs, respectively, trigger and reduce thrombogenesis (for review, see [69]). For instance, PAR-4 activation [80] and PAR4-activating peptides [143] trigger platelet aggregation; consistently, PAR antagonism attenuates platelet activation/aggregation (for details, see Section 10.6) in line with such inflammation-dependent thrombogenesis. An earlier study has shown that *P*-selectin causes leukocyte accumulation to facilitate fibrin deposition [144], complementing thrombotic episodes. P/E/L-selectins, ICAM, and VCAM are responsible for leukocyte adhesion/rolling/recruitment interacting with platelets and VEC to enhance thrombus formation [145]. In parallel, selectin-deficient mice lacking the activation of the extrinsic pathway are defective in fibrin production [140]. Antibodies to cytokines and adhesion molecules attenuate venous thrombosis [146]. LYP20, an antibody against *P*-selectin, blocks leukocyte adhesion to EC and platelets [147] and modifies thrombosis [148], and *P*-selectin inhibition decreases vein wall fibrosis [149]. 

In addition, there is a general perception of inflammation-dependent thrombogenesis, which is supported by the observations that anti-inflammatory agents are of antithrombotic benefits. For instance, nonsteroid anti-inflammatory drugs readily block thrombosis. COX-1 inhibitor such as low doses of aspirin suppresses platelet aggregation [150]. Similarly, COX-2 inhibition downregulates VEC/leukocyte activation [151].

### 7.2. The Circuit

Thrombosis-inflammation connection (Figure 3(4)) is integrated into the coagulation-inflammation vicious cycle (Figure 3(2) and (3)), thus rounting a complete circuit to link among coagulation, inflammation, and thrombosis. Concomitant with suppressed TF expression by COX inhibitors [35–37], the anti-inflammatory and antithrombotic properties of COX-2 inhibitors [150, 151] seem likely to be in agreement with the involvement of TF hypercoagulability in driving the coagulation-inflammation-thrombosis circuit. Further, activated platelets stimulate TF expression [152], while antiplatelet agent (dilazep) inhibits TF expression [153]. Both observations are in favor of the thrombosis-inflammation connection (Figure 3(4)) being part of the operative blood coagulation-inflammation-thrombosis circuit. The paradigm has also been observed in lung [154] and inflammatory bowel syndrome [155] while closely relating to cardiovascular risks [9, 69]. 

## 8. Coagulation-Dependent Events: Thrombosis/Inflammation-Associated Conditions

Mounting evidence reveals that TF hypercoagulability plays pathogenic roles closely relating to its not only inflammatory but also thrombotic actions. By driving the circuit (Figure 3), TF hypercoagulability is readily involved in an array of metabolic syndromes (e.g., atherosclerosis, hypertension, diabetes II, and obesity) and other clinical manifestations (e.g., cancers, antiphospholipid syndrome (APS), and fetal loss). 

### 8.1. TF in Sepsis/DIC

Hypercoagulation is often observed in septic shock including endotoxemia or systemic inflammatory responses after trauma, which mainly results from TF overexpression [156–158]. The ability of TF blockade to ease septic shock [83] or organ injury [159] points to a fundamental pathogenic role of TF in sepsis. Extrinsic infection/inflammation upregulating TF expression mediates enormous local or systemic intrinsic inflammation as well as a thrombotic condition via the operational circuit (Figures 2 and 3). A common manifestation presents DIC, an acquired disorder with hemostatic imbalance; excessive FIIa formation leads to fibrin deposition in microcirculation and consequent ischemic organ damage. Thus, such autocrine or paracrine TF signaling could lead to substantial tissue damages or multiple organ failure. 

### 8.2. TF in Cancers

TF overexpression has been reported in ovarian cancer [160], endometriosis [161], breast cancer [162], nonsmall cell lung carcinoma [163], prostate cancer [164], pancreatic cancer [165], melanoma [166], colorectal cancer [167], gastric cancer [168], esophageal cancer [169], hepatocellular carcinoma [170], brain tumor glioblastoma [171], leukemia [172], and lymphoma [173]. Accordingly, TF overexpression could be considered a biomarker for solid tumors [174].

The roles of TF in cancer have been demonstrated with severalfold relevance in relation to thrombotic condition, tumorigenesis per se and TF signaling (i.e., coagulation-dependant inflammation). Cancer linked with hypercoagulability and thrombotic risk has long been recognized by Armand Trousseau since 1865. The American Society of Hematology calling for a special session on “cancer and thrombosis” addresses its complex clinical interface of prothrombotic association with malignancies and prophylactic approaches. Cancer certainly could be recognized as a prothrombotic risk factor, leading to, for instance, venous thromboembolism and its complication of pulmonary embolism and mortality. Namely, cancers readily induce thrombosis [175]. Enhanced TF expression typically accounts for the mode of mechanism of thrombosis accompanied by suppressed TFPI [176] and defective APC anticoagulation system. Not only tumor cellular membrane-bound TF, but also microparticle-associated TF [177] links cancer to thrombosis. In addition, the similar hypercoagulable state exists in cancer stem cells [178]. 

The critical role of TF in tumorigenesis is supported by the observations that inhibited TF expression blocks tumor growth, metastasis [179], angiogenesis [180], cell invasion [181], and many other cancer characteristics. TF per se plays important roles in cell proliferation, tumor development, and progression apart from the accompanying coagulation-dependent inflammatory environment including MMP-9 [182], growth factors (VEGF, EGF, PDGF, etc.), and adhesion molecules certainly promoting “autocrine” tumorigenesis. Either VEGF or EGF in trun stimulates sustained TF expression [27, 28]. PTEN loss and tumor hypoxia readily induce TF expression [183], which could highlight TF as a major player in cancer progression. 

Tumor-expressed TF promotes growth by increasing cell survival and/or angiogenesis. TF and VEGF expressions mutually enhance each other [184], where VEGF is a known main angiogenic factor of cancer characteristics. TF cytoplasmic domain has been shown to be critical for VEGF expression [185]; conversely, VEGF causes TF promoter activation and involves gene upregulation with transcription factor NFAT involvement [28]. It is of particular interest to note that the serine phosphorylated cytoplasmic domain inhibits cellular cytotoxicity [186], thereby leading to increased tumor survival and metastatic rate. In addition, increased TF cytoplasmic domain phosphorylation and PAR-2 activation significantly correlate to cancer relapse [181]. Thus, a cooperation of the phosphorylated TF cytoplasmic domain with protease signaling could account for diverse contributions of TF to metastasis and angiogenesis [81, 187]. 

As the proceeding of TF-initiated extrinsic pathway, the resulting FIIa generation and fibrin production are of proangiogenesis. Furthermore, TF/FVIIa activates BcL2 [188], and FXa inactivates caspase-3 [189], both of which inhibit apoptosis. TF/FVIIa/FXa ternary complex possibly mediated by PAR-1/2 readily induces Erk1/2, Akt/PKB, and mTOR activation, all of which enhance the downstream signaling target phosphorylation for cancer cell undergoing antiapoptosis [190] and cell migration [191]. FIIa-PAR signaling in metastasis [192]/angiogenesis [94] and TF/FVIIa/PARs signaling in tumor growth [193] are also evident. FIIa could be recognized as a tumor growth factor [192, 194], which is accompanied by the enhanced tumor cell cycle mediated by downregulation of p27Kip1 and upregulation of Skp2 and MiR-222 [195]. FIIa is also able to upregulate cathepsin D which enhances angiogenesis, growth, and metastasis [196]. FIIa activates fibrinolysis inhibitors (e.g., TAFI [74] and PAI-1 [76]), further promoting cancer progression [197].

### 8.3. TF in Obesity

TF gene overexpression in obese has been reported for more than a decade [198, 199] accompanied by upregulated PAI-1, angiogenesis, cell adhesion, and so forth, all of which could stem from TF hypercoagulability. Inflammation has been proposed to engage in obesity development [200], while less is clear about the precise role of thrombosis per se in obesity. With the functional coagulation-inflammation-thrombosis circuit (Figure 3), triggered inflammation constitutes the pathogenesis of obesity with manifestation including diabetes and cardiovascular risks (e.g., atherosclerosis, hypertension). 

TF signaling (Figure 2) sets up inflammation, in part well accounting for elevated levels of IL-6 [200, 201], IL-8 [201], and TNF*α* [200] detected in obese subjects. Among which, either local or systemic inflammation (TNF*α*) significantly contributes to obesity [202]. Based upon high leptin and low adiponectin levels in obesity, the ability of leptin [30] or adiponectin [47], respectively, to augment or suppress TF synthesis could imply a mechanistic role of TF in developing inflammatory obesity. Furthermore, the involvement of PPAR*α* agonists [52]/activators [53] in downregulating TF expression also likely underlines a positive TF function in the process of inflammatory obesity. 

### 8.4. TF in Diabetes

Diabetes including type I and II is a hypercoagulable state [203] with elevated plasma levels of clotting factors (FVII, FVIII, FX, FXI, FXII), D-dimers, and TAT accompanied by decreased AT III, heparin cofactor II, or APC, presenting a thrombotic condition. 

Under hyperglycemia, excessive plasma glucose nonenzymatically conjugates with plasma proteins (e.g., hemoglobins) to form AGE. AGEs through their receptors exhibit biological damage in various tissues such as renal failure and vascular complications. For instance, hyperglycemia induces damage to vascular endothelial cells, which is mediated by the complex activation of MAPK, PKC, NF-*κ*B, and ICAM-1, primarily causing hemostatic alterations [204]. 

Increased circulating AGEs enhance TF expression [205], making diabetes a hypercoagulable and thrombotic condition [203, 205, 206]. Platelet TF in diabetes II appreciably increases [207]; increased FIIa and FXa generations are also found in diabetic platelets, enhancing the thrombotic nature. TF overexpression essentially promotes diabetes progression as well as its manifestation. As a consequence of diabetic TF hypercoagulability, elevated inflammatory mediators elicit cardiovascular complications including atherosclerosis. It is estimated that 80% diabetic patients die from a thrombotic disease and 75% of which result from cardiovascular complications [208]. Diabetic complications are more threatening than hyperglycemia *per se;* accordingly, relief of hypercoagulability could become far more important than glycemic control. Population-based clinical trials (ACCORD [209], ADVANCE [210] as well as VADT [211]) have demonstrated no benefit to cardiovascular risk in diabetes II upon glycemic control with significant low AGE (e.g., glycated hemoglobin <6%) for 2–3.5 years. Furthermore, rosiglitazone substantially lowering glycemia surprisingly increases the risk of myocardial infarction and death from cardiovascular causes [212]. For diabetic cardiovascular events, one could not expect that glycemic control per se significantly and promptly reverses the downstream damages done by AGEs. Apparently, nonglycemic factors (e.g., hypercoagulability, hypertension, and hyperlipidemia) play important roles in such complications. 

Apart from thrombotic natures, TF could assume a pathogenic role in diabetic progression in a close relation to inflammatory process [213, 214]. It is likely that TF signaling (Figure 2) through the coagulation-inflammation-thrombosis circuit (Figure 3) operating in diabetes could well be responsible for insulin resistance. Proinflammatory mediator TNF*α* is known to promote insulin resistance in which serine phosphorylation of insulin receptor substrate (IRS) is encouraged. As a consequence of preventing insulin downstream IRS tyrosine phosphorylation, TNF*α* thereby blocks insulin signal transduction [215]. In sharp contrast to TNF*α* negative effects on insulin action, adiponectin positively enhances insulin sensitivity, and hypoadiponectinemia accordingly leads to insulin resistance [215]. Notably, anti-inflammatory adiponectin suppresses TF expression [47], which could be in support of the role of TF in diabetes pathology. From the viewpoint of PPAR*α* activation improving insulin sensitivity, the observations of PPAR*α* agonists [52]/activators [53] downregulating TF expression also likely point to positive TF function(s) in insulin resistance involving inflammatory diabetes development. 

In summary, TF function has twofold significance in diabetes. TF not only dictates diabetic hypercoagulable nature and thrombotic outcomes [203], but also overlays its signaling in proinflammation (Figure 2) for insulin resistance. The ability of insulin [40] or an antidiabetic agent (metformin) [59] to attenuate TF expression seemingly reinforces a key pathogenic role of TF in diabetes.

### 8.5. TF in Cardiovascular Complications

Cardiovascular complications are a group of disorders closely associated with either inflammation or thrombosis or both. In these regards, it is not surprising that TF plays a major role in their pathogeneses [9]. TF overexpression, often correlated to gain-of-function of TF promoter polymorphism (A603G), promotes the development of cardiovascular diseases [216]. It has long been established that TF participates in the phase III of plaque rupture [217] during atherogenesis. TF expression is upregulated in atherosclerotic plaques of patients with unstable angina and myocardial infarction [218]. 

TF hypercoagulability driving the coagulation-inflammation-thrombosis circuit (Figure 3) readily extends its diverse consequences to cardiovascular complications and vascular diseases [219] including arrhythmias [58], arterial hypertension [220], hypertrophy [221], ACS [222, 223], andatrial fibrillation (AF) [224], TF hypercoagulability with elevated proinflammatory cytokines (Figure 2) could in part well contribute to atherosclerosis known as chronic inflammatory disease [217]. In cultures, recombinant TF induces cellular apoptosis with increased caspase-3 activity and nuclear location of p53 while increasing cellular proliferation/hypertrophic growth [221]. As a consequence of accelerated cardiomyocyte turnover, TF could contribute to the induction and progression of cardiac hypertrophy. Angiotensin II stimulates TF synthesis [220], mediating hypertensive action. Histamine augments TF expression, accounting for its action in ACS [225]. In conjunction with its effects on endothelial damage/dysfunction and angiogenic actions [224], TF upregulation could well be involved in a thrombogenic state of AF [224].

In contrast, TF deficiency in mice shows cardiac fibrosis [226, 227] largely based upon TF functions in normal extracellular cardiac homeostasis, extracellular matrix regulation, and vascular maintenance [227]. Apparently, cardiac bleeding/hemorrhages in TF deficiency certainly encourages its fibrosis [226] where PA involvement could also be ensured by insufficient FIIa generation [74–76]. It awaits further confirmation in human conditions.

### 8.6. TF in Autoimmune Disorder: Antiphospholipid Syndrome (APS)

Classically, APS is generally characterized by the presence of aPL including lupus anticoagulants, anticardiolipin antibodies, and anti-*β*2-glycoprotein-1 (*β*2GPI) antibodies. It is proposed that TLR-4 mediates anti-*β*2GPI-induced TF expression [228]. Alternatively, TF overexpression results from APS-associated complement activation. aPL activates complement via the classical pathway; activated complement (e.g., C5a) drastically stimulates TF synthesis. This autoimmune thrombophilic condition is largely due to enhanced coagulation (e.g., TF overexpression) accompanied by attenuated downregulation of blood coagulation (e.g., inhibited APC, TFPI, and AnxA5) and suppressed fibrinolysis. Increased microparticles and TF expression are found in APS with prothrombotic conditions of various manifestations, most commonly venous and arterial thromboembolism and recurrent pregnancy loss. 

In addition, TF could play a pathological role in APS manifestation. It is not surprising if APS of TF overexpression also presents a hyperinflammatory condition in view of the paradigm of coagulation-inflammation-thrombosis circuit (Figure 3). Apart from that aPL-induced complement activation contributes to inflammation [229], TF signaling could well account for increased TNF*α* [230–232] production as major proinflammation reported in APS patients [233] in which TNF*α* also seems to be responsible for its manifestation: fetal damage [232].

### 8.7. TF in Miscarriage

Miscarriage including fetal death, preeclampsia, and intrauterine growth restriction often closely links to APS involving complement and angiogenic actions. During trophoblast differentiation, aPL activates complement via the classical pathway. Complement activation (C3 and C5a) directly mediates placental injury and causes fetal loss and growth restriction, resulting from an imbalance of angiogenic factors (e.g., VEGF and placental growth factor) as well as their corresponding receptors that are required for normal placental development [234].

Alternatively, TF overexpression is triggered by aPL-induced complement activation, and TF signaling fulfills such miscarriage/placenta damage/fetal injury. For instance, recent research demonstrates that neutrophil activation by TF/FVIIa/PAR-2 signaling [235] mediates aPL-induced pregnancy complication. In fact, TF on neutrophils and monocytes is a critical mediator in trophoblast injury and embryo damage in aPL-dependent or independent pregnancy loss [236]. Rapid increases in decidual and systemic TNF-*α* level are also responsible for fetal death/loss [232], which could be in line with TF signaling (Figure 2) playing a pathogenic role. Further, FIIa-induced platelet activation/aggregation activates complements, possibly conferring the direct fetal damage [234]. 

Anti-TF mAb prevents aPL-induced pregnancy loss [234], while statins [237, 238] may be a good treatment for women with recurrent miscarriages and intrauterine growth restriction. These clinical studies are consistent with a pathogenic role of TF in APS-induced fetal damage.

### 8.8. TF in Wound Healing

Wound, including diabetic foot, healing process generally consists of three phases (inflammatory, proliferative, and remodeling phases) that continuously overlap one another during the process. Hemostasis initiates angiogenesis-dependent wound healing. TF overexpression often occurring after wounding, trauma, or surgeries in part accounts for hypercoagulability encouraging wounding healing [239–241]. Given that inflammation involved in the initial phase, such “autocrine” or “paracrine” TF signaling essentially ensures fibrin matrix formation, angiogenesis, production of growth factors (VEGF, PDGF, bFGF, TGF*α*/*β*, etc.), adhesion molecules, and so forth, (Figures 2 and 3), all of which significantly contribute to wound healing process.

### 8.9. TF in Development

Limited evidence reveals that TF extracellular domain is essential for embryogenesis [242–244], which is believed to be mediated by TF-dependent FIIa generation and PAR-1 activation. Thus, TF serves as an important morphogenic factor during embryogenesis. Apparently, TF signaling with FVIIa, FXa, and FIIa generation for PAR activation/transduction triggers an array of biological events as a consequence of proinflammation (Figure 2, right panel), among which growth factors (EGF, VEGF, PDGF, bFGF, etc.) could play major roles in development [245]. Consistently, inactivation of TF gene results in embryonic lethality in a murine model [243]. It is said that TF expression coordinated with TFPI, ATIII, and FVII levels could be critical in embryonic development [246].

### 8.10. TF in Other Diseases

TF expression is often upregulated by an antibody to platelet factor 4 (PF4) upon a long exposure/treatment of heparin [247]. In heparin-induced thrombocytopenia, PF4 also impairs APC activity, making a pronounced hypercoagulable and prothrombotic condition. TF overexpression in adult onset asthma significantly correlates to the gain-of-function of TF promoter polymorphism (A603G) [216]. 

Concerning innate immunity and acute inflammation, complement activation is of TF relevance. Complement activation, especially C5a, upregulates TF expression, thereby extending to a broad spectrum of immune consequences [248]. TF overexpression exhibits “paracrine” signaling for fulfilling innate immunity regardless of TF expression by neutrophils remaining debatable. Similarly, TF overexpression is observed in bacterial (pneumonia [249], *Helicobacter pylori* [250]), viral (HIV) [251], or parasite (malaria) [252] infection.

In response to surgical procedures, enhanced TF synthesis is reported in major surgeries such as hip replacement, cardiopulmonary bypass (CPB) [253] or transplantation [254–256]. Upon tissue injury, exposure to protein disulfide isomerase and PS readily activates TF [10–12] and its signaling. It is plausible that TF hypercoagulability in part accounts for postsurgical inflammatory responses.

With regard to lifestyles, smoking upregulating TF expression apart from its apparent free radical inhalation elicits diverse health problems including cardiovascular and cancer risks. High-fat diets [257], oral contraceptives [15, 258], and estrogen replacement [21, 259] also promote TF expression, possibly driving the circuit (Figure 3) for diverse clinical manifestations in relation to inflammation or/and thrombosis. 

In addition, TF overexpression is associated with other pathological conditions such as liver cirrhosis [260], synovial inflammation [261], sickle cell anemia [262], or hepatic necrosis during cholestasis [263]. These pathological conditions likely result from the coagulation-inflammation-thrombosis circuit (Figure 3); the precise mechanisms of action however remain to be defined.

## 9. Noncoagulation-Mediated TF Roles

The signaling function of TF cytoplasmic domain has been demonstrated although its biochemical mechanism remains unclear. For instance, cross-talk between intracellular TF domain with integin *α*3*β1* promotes cell migration [264], while the cytoplasmic domain possibly upon phosphorylation of the three serine residues causes hyperchemotaxis [265]. The cytoplasmic domain contributes to renal albumin retention, and its renal expression protects against proteinuria. Consistently, the absence of the cytoplasmic domain is associated with increased albuminuria, increased spontaneous glomerular TNF*α* production, podocyte effacement/inflection, reduced podocyte numbers, resulting in albuminuria and proteinuria [266]. For cancer progression/relapse, increased cytoplasmic domain phosphorylation significantly correlates to metastasis and angiogenesis [267]. It is proposed that the cytoplasmic domain per se is critical for VEFG expression [185], an important angiogenic component in tumorigenesis. 

## 10. Antagonisms against TF Signaling-Evolving Thrombotic or Inflammatory Events

In view of the paradigm of coagulation-inflammation-thrombosis circuit (Figure 3), any interruption of the circuit is accordingly expected to exert broad antagonism against hypercoagulation, inflammation, thrombosis, and their complications.  Table 1 lists some typical examples of targeting TF hypercoagulation for fighting diverse pathological conditions in cell cultures, *ex vivo*, animal studies, or clinical trials. Strategies targeting TF signaling include TF suppression, general anticoagulation, FVIIa inhibition, FXa inhibition, FIIa inhibition, PAR antagonism, and many others. 

### 10.1. TF Suppression

Inhibited TF synthesis readily leads to many clinical applications for easing pathological conditions including inflammation, thrombosis, and cardiovascular dysfunctions. For instance, vitamin D3 deficiency often exists in APS; consistently, vitamin D3 inhibits transcription factors (e.g., AP-1 and NF*κ*B) to reduce TF overexpression for easing APS-induced thrombosis [268]. 1, 25(OH)_2_ D3 analogs are also used for immunomodulation and antineoplastic therapy of leukemia [50]. A novel NO-releasing statin derivative exerts antiplatelet/antithrombotic activity [269]. Indobufen, through a thromboxane-mediated mechanism, exhibits antagonisms against atherothrombosis [57]. Amiodarone inhibiting TF translation attenuates arterial thrombosis including coronary artery thrombosis as much as ventricular arrhythmias [58]. Nicotinamide inhibits coagulation and inflammation, resulting in anti-inflammation with reduced IL-6 and CD11a in sepsis or DIC [41]. ACE inhibitors offsetting ATII-induced TF overexpression reduce the risk of recurrent myocardial infarction in patients with left ventricular dysfunction [46]. Ethyl pyruvate inhibiting TF mRNA expression shows combined anti-inflammatory and anticoagulant effect [44]. DMSO inhibiting thrombus formation and vascular smooth muscle cell activation could improve acute coronary syndromes [45]. Liver X receptor agonists attenuate atherothrombosis [54]. A hairpin ribozyme inhibiting TF gene expression and TF mRNA shows antithrombotic action [63]. Hydroxyurea has antithrombotic activity [43], while pentoxifylline attenuates DIC [55]. Adiponectin could prevent endothelial dysfunction and atherogenesis in acute coronary syndrome [47]. PPAR*α* agonists [52]/activators [53] reduce the thrombogenicity of atherosclerotic plaques. TF suppression by adiponectin [47] or PPAR*α* activation [52, 53] could also constitute antagonism against diabesity. Metformin, an antidiabetic agent, suppresses the production of TNF*α* [59], a known factor for insulin resistance [213–215]. Antisense oligonucleotide blocking TF expression prevents leukocyte adhesion following renal ischemic reperfusion injury [66, 270]. COX inhibitors readily show anti-inflammation [152, 153] as well as antithrombosis. Red wine phenolics and quercetin improve cardiovascular health and prevent CHD [56]. Guggulsterone suppresses TF expression together with anti-inflammation and antagonism against arterial thrombosis [120]. HMG-CoA reductase inhibitors (e.g., pravastatin) prevents APS-mediated miscarriages and placental and fetal injury [33, 34, 237, 238], in addition to the general anti-inflammatory effects of statins on lowering CRP, IL-1*β*, IL-6, and so forth. However, little is known and remains inconclusive about the antithrombotic/anti-inflammatory relevance of targeting TF synthesis by various inhibitions of intracellular signaling kinases (e.g., MAPK, PKC) or transcription factors (e.g., NF*κ*B); the signaling downregulation per se already shows anti-inflammation [4].

Interestingly, paclitaxel exhibits anticancer activity [38]. COX-2 inhibitors show the prevention of colorectal cancer [271], while all-trans retinoic acid inhibiting cancer procoagulation could of benefit to leukemia [49]. shTF RNA inhibits breast cancer growth/angiogenesis *in vivo* independent of VEGF regulation in mice [62], and TF RNAi antagonizes metastasis [272]. 

### 10.2. FVIIa Inhibition

FVIIa inhibition readily shows antagonism against inflammation. Recombinant nematode anticoagulant protein c2 (NAPc2), a novel inhibitor for TF/FVIIa complex, diminishes coagulation-dependent IL-6 and IL-8 productions [87]. Active site-inhibited FVIIa depresses LPS-inducible plasma levels of TNF-*α* [273], IL-6 [273–275], and IL-8 [274, 275]. FVIIai suppresses sTF-induced inflammation in an *in vivo* model [80]. A small molecule BCX-3607 (TF/FVIIa inhibitor) also decreases IL-6 level in an endotoxemia mouse model [276].

Hemextin AB complex, a snake venom protein complex, directly inhibits FVIIa catalytic activity for anticoagulation [277]. Active site-blocked FVIIa [278] and BMS-593214 [279] provide cardioprotection and carotid arterial and venous thrombosis. Bolus of FFR-rFVIIa reduces thrombus and fibrin deposition in A-A shunt rat model [280]. FFR-rFVIIa inhibits *ex vivo* fibrin deposition in patients undertaking percutaneous coronary intervention [281]. DEGR-rFVIIa prevents thrombus formation in whole blood [282]. Similarly, an active site-blocked FVIIai attenuates fibrin/platelet deposition [283]. By altering TF/FVIIa binding and inhibiting its activity, sTF mutant reduces arterial thrombosis in guinea pigs [284]. A cyclic dodecapeptide (PN7051) derived from the second EGF-like domain of FVII interferes with TF/FVII/FX complex to attenuate fibrin deposition, platelet-fibrin adhesion and platelet-thrombus formation [285]. PHA-798 diminishes thrombus formation in primates [286]. It remains to be determined concerning the antithombotic application of rNAPc2.

Remarkably, it has also been documented that FVIIa inhibition exhibits anticancer actions. rNAPc2 [287, 288] or active site-blocked FVIIa [289] inhibits cancer metastasis, angiogenesis, and/or tumor growth. 

### 10.3. FXa Inhibition

A growing list of oral FXa inhibitors is developed and available; animal or clinical studies show therapeutically anti-inflammatory applications: LMWH, enoxaparin, or DX9065a suppressing *P*-selectin, TNF-*α*, IL-6 [290], or MCP-1 [291] expression. ZK-807834 attenuates FXa-induced IL-6 production [89]. LMWH (AV 526 [292]) and direct FXa inhibitors (biarylmethoxy isonipecotanilides [293]) are antagonistic against AT/VT and coagulation. LMWHs including Fondaparinux [294], Enoxaparin [295], Bemiparin [296], Tinzaparin [297], Fraxiparine [298], Reviparin [299], and Dalteparin [300] exhibit clinical benefits for arterial/venous thrombosis, venous thromboembolism (VTE), and DVT; all LMWHs are able to markedly inhibit platelet aggregation in whole blood. SamOrg 123781A has recently been evaluated for its antithrombotic application with reduced platelet adhesion and thrombus formation in pigs [301]. Recombinant antistasin (rATS) or tick anticoagulant peptide (rTAP) reduces restenosis in balloon angioplasty rabbits [302], and rTAP reduces TF/FVIIa-dependent thrombus formation *in vitro* [303]. DX-9065a depresses platelet aggregation [304] and leukocyte adhesion to EC [305] while providing effective protection against tumor-induced DIC [306]. Newly developed TAK-442A shows antithrombotic and anticoagulant activities against venous thrombosis [307]. Orally active amidinoaryl propanoic acid reduces platelet deposition and fibrin accumulation in venous-type thrombus in baboons [308]. ZK-807834 inhibits arterial thrombosis [309] as well as venous thrombosis in vascular injury rabbits [310] and electrolytic injury canines [311]. SF 303 and 549 inhibit A-V shunt-induced thrombus formation in rabbits [312]. Orally active YM-75466 inhibits thrombosis in mice [313]. FXV673 inhibits thrombus formation in canines [314]. Orally active pyrazole DPC423 attenuates electrically induced carotid artery thrombosis in rabbits [315]. Isoxazolines and isoxazoles prevent A-V shunt thrombosis [316], while RPR120844 reduces venous thrombosis in rabbits [317]. Rivaroxaban prevents and treats venous thromboembolism and is used for stroke prevention in AF [318]. GW813893 is of antithrombotic therapeutic benefits [319]. Apixaban inhibits platelet aggregation [320]. DU-176b is considered a new anticoagulant for the prophylaxis and treatment of thromboembolic diseases [321]. Oral BAY 59-7939 is for the prevention of venous thromboembolism [322]. Many more direct FXa inhibitors await clinical studies for their anti-inflammatory and antithrombotic applications.

Anticancer activity through direct FXa inhibition is also reported. WX-FX4 effectively inhibits metastasis/tumor growth/angiogenesis and prolongs survival [323]. LMWH Tinzaparin shows antimetastatic effect [324]. Ixolaris is able to block primary tumor growth and angiogenesis [325]. DX-9065a inhibits cell proliferation [326], and MCM09 shows anticancer action by significantly lowering lung metastasis [327]. 

### 10.4. FIIa Inhibition

Heparin shows a variety of anti-inflammatory potentials (for review, see [328]). Heparin-bonded circuit prevents the increases in IL-6 and IL-8 in CPB patients [329], while heparin bolus reduces neutrophil activation without affecting platelet aggregation [330]. Heparin is also considered a treatment for pregnancy loss [331]. 

Direct FIIa inhibitor (hirudin) binds to FIIa active site and prevents PAR-1 from cleavage [332], thereby diminishing FIIa signaling in ICAM/VCAM expression [96] and elicitation of VEGF [333, 334], IL-6 [139], IL-8 [93], or MCP-1 [93]. Hirudin suppresses sTF1-219-induced inflammation [80]. A hirudin analog (lepirudin) alleviates LPS-induced platelet activation [335]. Lepirudin, desirudin, and bivalirudin [336] exhibit antagonism to DVT, VTE, and arterial thrombosis in clinical studies. 

FIIa active site inhibitor (melagatran) diminishes *P*-selectin expression [332], ximelagatran [337] shows various antithrombotic actions, and argatroban attenuates DVT and VTE [338]. Org 42675 is a direct anti-FIIa agent with anti-FXa activity, seemingly being superior to argatroban and fondaparinux in animal models of thrombosis [339].

A new direct FIIa inhibitor (FM-19) shows platelet inhibition in vitro and *in vivo* with an application for fighting ACS [340]; this oral anticoagulant also inhibits prostate tumor growth *in vivo* [341]. Several other direct FIIa inhibitors (e.g., argatroban [342], foypan [343], and dabigatran etexilate [344]) show promising anticancer potentials by preventing and slowing down tumor cell migration, metastasis, and cancer progression. Heparin and dalteparin downregulate PAR-1 cleavage [332], blocking PAR-1-mediated VEGF release in response to FIIa [93]. Heparin also reduces lung metastasis [327].

### 10.5. By Natural Anticoagulants: TFPI, APC, or ATIII

TFPI, a multifunction anticoagulant with trivalent Kunitz-type domains, downregulates TF-dependent blood coagulation by inhibiting FXa and TF/FVIIa complex. The first domain is responsible for the inhibition of FVIIa in TF/FVIIa complex by a feedback inhibition through the inactive quaternary complex TF/FVIIa/TFPI/FXa, where FXa accelerates TFPI binding to FVIIa. The second domain directly binds and inhibits FXa. APC directly inactivates FVa and FVIIIa. FVa is an essential cofactor for FXa (prothrombinase) in prothrombin activation, while FVIIIa functions as a high-affinity receptor/cofactor for FIXa (intrinsic Xase) in FX activation. AT III virtually inhibits all clotting factors at a slow rate; it mainly targets FIIa, FXa and FIXa. In addition, AT III complex with FVIIa inactivates FVIIa activity; the inhibition is enhanced in the presence of TF or heparin.

#### 10.5.1. Anti-Inflammatory Actions

TFPI plays a significant role in protecting against septic shock induced by *E. coli* in animal models [345], suppressing TNF-*α* expression and IL-6 and -8 production. TFPI suppresses coagulation-dependent IL-8 production [346] or VCAM-1 expression [347]. In cell cultures, TFPI reduces the autocrine release of PDGF-BB, MCP-1 and MMP-2 in response to FVIIa, and FXa [348]. Its coagulation-independent action includes the direct suppression in TNF-*α*, IL-6, and IL-8 production [349], reducing mortality from *E. coli* septic shock in baboons. TFPI also directly interferes with LPS reception [345]. TFPI in place of antibiotics could be a treatment for pneumonia [350]. Gene therapy with rTFPI could attenuate pulmonary fibrosis [351]. TFPI could also be used to relieve rheumatoid arthritis (RA) synovial inflammation [261]. 

It has long been established that APC protects from sepsis, DIC, and endotoxemia [352, 353]; APC is recognized as one of the effective anti-inflammatory agents in clinical applications. APC inactivates the production of IL-1, -6, -8 or TNF-*α* [354]. APC consistently reduces septic mortality and blocks DIC upon *E. coli*. infection in either animal or human models [355, 356].

 ATIII blocks FXa-induced IL-6, IL-8, MCP-1, ICAM/VCAM, and E-selectin expressions [90] in addition to arresting FIIa-induced (PAR-1-dependent) VEGF release [93] and MCP-1 expression [89]. ATIII inhibits LPS-induced IL-6 production [138]. Apart from inactivating NF*κ*B [357], AT III direct anti-inflammatory action includes the suppression in INF-*γ* and ILs (e.g., 1, 2, 4, 6, and 8) production, which is mediated by enhanced PGI production and diminished inducible NOS [358]. However, a discrepancy exists concerning improved survival rate in baboons [359] but not severe human sepsis treated with the high dose of ATIII [360]. Further research warrants verifying its anti-inflammatory action(s). 

#### 10.5.2. Antithrombotic Actions

rTFPI exhibits antithrombotic effect in a human *ex vivo* thrombotic model [361] without protection from dealth though, while a truncated TFPI 1-161 reduces thrombus formation [362]. 

APC antithrombotic potential is implied by increased APC resistance [363] and the deficiency [364] or low plasma level [365] of APC observed in thrombosis. APC profibrinolytic effects by inactivation of PAI-1 [366] and TAFI [367] synergistically diminish the direct thrombotic inputs from blood coagulation cascade. However, APC antithrombotic potential remains in the experimental stage of animal studies. For instance, a recombinant human APC (LY203638) inhibits arterial thrombosis in a canine model [368]. A human APC product (CTC-111) reduces venous thrombosis in mice [369]. FLIN-Q3 diminishes A-V shunt-induced thrombosis in guinea pigs [370]. hAPC attenuates rat mesenteric occlusion [371], and rhAPC inhibits arterial thrombosis in baboons [372]. Infusion of bovine APC suppresses thrombus formation in rats [373] and rabbit microarterial thrombosis [374]. A rabbit APC-loaded stent reduces thrombus and platelet deposition in vitro and *in vivo* [375]. 

Little is known about the antithrombotic application of AT III; a bolus infusion with ATIII attenuates FIIa-induced leukocyte rolling/adhesion/recruitment in ischemia/reperfusion [376].

#### 10.5.3. Anticancer Properties

The effects of TFPI, a “tumor suppressor-like molecule,” include enhanced apoptosis [377] and blocked tumor growth and angiogenesis [288]. TFPI-2 expression in tumor tissue could inhibit invasion, tumor growth, and metastasis [378]. ATIII demonstrates antimetastatic [379] and antiangiogenic potentials [380]. It remains unclear whether APC could exhibit consistent anticancer benefits [381] regardless of limited evidence showing inhibited tumor metastasis [382]. 

### 10.6. PAR Antagonism

PARs transmitting blood coagulation signals to cellular activation for proinflammation (Figure 2) are apparent therapeutical targets for interrupting the circuit (Figure 3). A growing list of PAR antagonists readily shows clinical applications concerning inflammation and thrombosis. For instance, RWJ 58259 [383] selectively blocks PAR-1, resulting in the attenuation in CD61 expression, platelet aggregation, thrombus formation, and restenosis. RWJ-56110 protects from FIIa-induced human platelet activation and platelet-mediated thrombosis [384]. Similarly, PAR-1 antagonists (SCH 79797 and 203099) depress *P*-selectin expression and platelet aggregation [385] and VEGF release [386]. SCH 79797 also limits myocardial ischemia/reperfusion injury in rat hearts [387] and offsets plasmin-induced IL-8 expression and PGE2 release [388]. Orally active himbacine-based SCH 530348 shows potent antiplatelet activity [389]. Refludan suppresses macrophage adhesion [390]. BMS 197525 [391] and 200261 [392] abolish platelet aggregation. Nonpeptide FR 171113 preferentially diminishes FIIa-induced thrombosis in guinea pig models [393]. TH146 and MAP4-TH146 readily inhibit FIIa-induced human platelet aggregation and mouse thrombosis [394]. 

By blocking PAR-2 activation, peptide antagonists (FSLLRY-NH_2_ and LSIGRL-NH_2_) suppress *Serratia marcescens* serralysin-induced IL-6/8 expression [109]. PAR2 mAb (SAM-11) and PAR2 antagonist (ENMD-1068) [110] significantly attenuate IL-1*β* production and joint inflammation. Anti-PAR-2 Abs and tryptase inhibitors (GW-45 and GW-61) cause significant decreases in IL-6 and IL-8 release from human peripheral blood eosinophils [108]. SR 48968 and 140333 reduce contractile [107]. FUT-175 consistent with PAR deficiency eases inflammatory bowel disease/symptom [395]. 

PAR4 antagonist (P4pal-10) is used for treatment of thrombocytopenia and DIC [396] protecting from systemic inflammation accompanied by stabilized liver, kidney, and lung function. A nonpeptide PAR-4 antagonist (YD-3) selectively depresses GYPGKF-induced platelet aggregation [397]. tc-Y-NH(2) and P4pal10 [398] provide protection against injury from myocardial reperfusion injury. P4pal-10 also protects from platelet-mediated thrombosis [399]. 

Similarly, general PAR downregulation could also achieve such anti-inflammatory and antithrombotic effects. For instance, IL-4 suppresses PAR-1, -2, and -3 mRNA expressions [400]. Cathepsin G and neutrophil elastase facilitate the internalization of PAR-1 [401]/-2 [402] to desensitize/disarm the reception function. The ubiquitination of PAR-2 by *β*-arrestin attenuates PAR-2 signaling induced by trypsins, tryptase, and coagulation mediators (FVIIa and FXa) [403]. By increasing GTPase activity of G_q?_, NO donors and cGMP [404] terminate PAR-1 signaling and exhibit vascular smooth muscle relaxation.

Concerning anticancer potentials, recent research advances reveal that PARs play roles in cancer metastasis [405] and angiogenesis [406]. Consistently, reduction of PAR-1 expression by siRNA or PAR-1 antagonism by SCH79797 significantly suppresses melanoma cell motility/invasion [406]. SCH79797 suppresses HIF and Twist expression attenuating cancer metastasis [407], while blocked VEGF release could be of antiangiogenesis [407].

### 10.7. Miscellaneous

Downregulation of TF function shows antithrombotic effects. An i.v. delivered antibody against rabbit TF (AP-1) inhibits intravascular thrombosis [408] and thrombus propagation without affecting bleeding time in rabbits [409]. Anti-TF mAb is of antisepsis [83] and prevents APS-mediated pregnancy loss [234]. TF blocking antibody (CNTO 859) readily reduces EGFR-mediated tumor initiation [178] and cancer initiation/angiogenesis [410]. TF blocking antibody also reduces allograft rejection [411]. Oral warfarin significantly reduces IL-6 at day 15 [412, 413]; this general anticoagulant also shows antagonisms against tumor growth/metastasis [414]. n-3 FA [25, 415], known as inflammation resolution, offsets AA stimulatory effect on TF expression [25] to ease inflammation and provide cardioprotection. 

## 11. Remarks

Blood coagulation, a primitive biological phenomenon in the animal kingdom, has historically been recognized as a host defense to prevent one from bleeding to death. TF-initiated extrinsic pathway, known as being inducible compared to constitutive intrinsic pathway, plays an integral role in blood coagulation, FIIa generation, and thrombus formation (for review, see [3, 10, 67]). Accumulating evidence demonstrates TF diverse biological effects in local or systemic inflammation [4]. Not only does the extrinsic pathway but also intrinsic pathway results in inflammation [416]. Interestingly, TF hypercoagulability refuels a coagulation-inflammation-thrombosis circuit in “autocrine” or “paracrine” fashion (Figure 3), thereby manifesting many pathological conditions.

Such extracellular TF signaling activates cells, and its pronounced effects include proinflammatory cytokine production (Figure 2). It has been elucidated that inflammasomal activation [417] in response to innate pathogens [418], viral [419], fungus [420], influenza [421], microbes [422], and chemicals (e.g., cholesterol [423], uric acid [424], or aluminium hydroxide [417] crystals, asbestos [425], silica [425]) is essential for cytokine secretion (for review, see [426]). It, however, remains elusive if inflammasomal activation is involved in such inflammatory process triggered by TF signaling. Thus far, there is no indication whether coagulant mediators (e.g., FVIIa, FXa, FIIa) could activate procaspase-1, facilitating proinflammatory cytokine secretion. Could PAR activation directly turn on inflammasomal activation, an interesting question seemingly further addressing the similar issues if inflammasomal activation is critical for coagulation-dependent inflammation?

Among diverse clinical conditions associated with TF overexpression and its signaling mentioned herein, the close link between TF hypercoagulability and neurological disorders is however seldom reported. Although high TF expression in the brain could in part account for thrombotic stroke consequences, it certainly warrants investigation to explore if TF and its signaling participate in other neuronal dysfunctions or CNS disorders. It would also be of particular interest to determine the biological events of coagulation concerning not only innate [248] but also adaptive immunity of B/T cell equipped and featured with such “autocrine” or “paracrine” TF signaling, if any.

In view of the paradigm of coagulation-inflammation-thrombosis circuit eliciting diverse pathological events (Figure 3), targeting TF hypercoagulation is of therapeutical relevance. Apparently, the development of anticoagulants is of broad pharmaceutical interests; anticoagulation could turn into strategic approaches for intervention and cure not limiting to thromboprophylaxis. It is highly promising that anticoagulants available arresting different stages of blood coagulation cascade [427] exhibit benefits other than hemostasis. Approaches to direct FVIIa, FXa, or FIIa inhibition readily demonstrate broad clinical applications (Table 1). In these regards, TF posttranslational downregulation (including encryption) could deserve attention for interventional therapeutical relevance in prospective of such upstream downregulation of the extrinsic pathway (Figure 2, left panel) with broad suppression of downstream proinflammatory coagulant mediators (e.g., FVIIa, FXa, and FIIa) as well as fibrin production.

The observations of anticoagulation exhibiting anticancer properties clearly demonstrate the new frontiers of the emerging therapeutical era. Direct PAR blockade could be part of therapeutically targeting coagulation-dependent inflammation and the circuit (Figure 3). Further research is needed to study if PAR antagonisms could widely exhibit an array of clinical benefits to relieve diseases including cancer, obesity, diabetes, APS, and others in addition to inflammation and thrombotic related cardiovascular complications.

Like any other therapies, anticoagulation bears certain limitations and cautions for its applications. For employing anticoagulants, bleeding or hemorrhage episodes become major concerns in relation to the safety and efficacy issues upon long-term uses or “over dosages.” With cautions in mind, routine monitoring for hemostatic properties is highly recommended. Further investigations warrant addressing rationally designed anticoagulant approaches to achieving/maintaining/weighing in therapeutical benefits for diverse clinical applications.

## Figures and Tables

**Table 1:** Targeting TF-initiated coagulation and signaling consequence for easing clinical events.

Strategy and agent	Antagonism against
**TF suppression**	
HMGCR inhibitors	Inflammation; thrombosis; miscarriage; APS; cancer growth
COX inhibitors	Inflammation, APS; thrombosis; miscarriage; cancers
Vitamin D3	APS; thrombosis; cancer
Amiodarone	AT; arrhythmia
Ethyl pyruvate	Inflammation; coagulation
NO	Platelet activation; thrombosis
Indobufen	AT
Hydroxyurea	Thrombosis
RNAi	Metastasis
DMSO	ACS
Adiponectin	Atherogenesis; diabetes; ACS
Hairpin ribozym	Thrombosis; I/R injury
TF sh RNA	Breast cancer growth/angiogenesis
Metformin	Diabetes II
Liver X receptor agonists	AT
Paclitoxel	Cancers
Antisense TF ODN	I/R injury
ACE inhibitors	MI
Nicotinamide	Sepsis; DIC; coagulation; inflammation
PPAR*α* activation	Obesity; diabetes
Guggulsterone	Inflammation; AT
ATRA	Leukemia; CHD
Phenolics/resveratrol	CHD

**FVIIa inhibition**	
BcX-3607	Thrombosis; inflammation
FVIIai	Colorectal metastasis; inflammation; thrombosis/MI
rNAPc2	Coagulation; inflammation; angiogenesis; tumor growth
Hemextin AB	Coagulation
BMS593214	AT; VT
PN7051	Thrombosis
PHA-798	Thrombosis
FFR-rFVIIa	Inflammation; thrombosis; metastasis

**FXa inhibition**	
Fondaparinux	DVT; VTE; PE
Enoxaparin	Inflammation; AT; VT
WX-FX4	Metastasis/tumor growth/angiogenesis
DX-9065a	Inflammation; platelet aggregation; DIC; tumor proliferation
TAK-442	Coagulation; VT
ZK-807834	Inflammation; AT; VT
Oral rivaroxaban	AF; VTE
Oral GW 813893	Thrombosis
Oral BMB344577	Cancer proliferation
Oral apixaban	Platelet aggregation
LMWH AVE5026	AT; VT; coagulation
Oral DU176b	Thromboembolism; coagulation
Oral sulfanilamide	Coagulation
Oral DPC423	Thrombosis
Oral YM-75466	Thrombosis
MCM09	Cancer metastasis
Oral BAY597939	Thromboembolism
Ixolaris	Tumor growth; angiogenesis
NAP5	Coagulation
Tinzaparin	Metastasis
SamOrg123781A	AT
rTAP (rAST)	Thrombosis; restenosis
Rivaroxaban	Stroke; AF

**FIIa inhibition**	
FM-19	Platelet activation; ACS; tumor growth
Dabigatran etexilate	Breast cancer progression
Argatroban	DVT; VTE; tumor migration/metastasis
Heparin	Inflammation; DVT; VTE; pregnancy loss; metastasis
Foypan	Metastasis
Ximelagatran	DVT; VTE; AT
Hirudins	Inflammation; DVT; VTE; AT
Org 42675	AT

**PAR blockade**	
SCH 7979	Inflammation; platelet aggregation; I/R injury; cancer cell motility/metastasis/angiogenesis
RWJ 56110	Platelet aggregation; thrombosis
RWJ 58259	Platelet aggregation; vascular occlusion; neointimal thickness; restenosis; thrombosis
PAR-1 antibody	Platelet aggregation
PAR-2 mAb	Joint inflammation
ENMD-1068	Joint inflammation
P4pal	DIC; thrombocytopenia; I/R injury
YD-3	Platelet aggregation
SFLLR	Platelet aggregation
FR 171113	Platelet aggregation; AT
TH146	Platelet aggregation; thrombosis
FSLLRY-NH2	Inflammation

**Miscellaneous**	
TFPI	Inflammation; pulmonary fibrosis; VT; pneumonia; RA; cancer; apoptosis
APC	Inflammation; AT; VT; sepsis; metastasis; apoptosis
AT-III	Inflammation; thrombosis; metastasis; angiogenesis
Dilazep	Platelet aggregation; APS
CNTO 859	Tumor initiation/growth/angiogenesis
Anti-TF mAb	Septic shock; DVT; AT/VT; miscarriage
Oral warfarin	Inflammation; thrombosis; metastasis; tumor growth
n-3 FA	Inflammation

APS: antiphospholipid syndrome; ACS: acute coronary syndromes; AF: atrial fibrillation; ATRA: all-trans retinoic acid; AT: arterial thrombosis; CHD: coronary heart disease; COX: cyclooxygynase; DIC: disseminated intravascular coagulation; DVT: deep vein thrombosis; HMGCR: HMGCoA reductase; MI: myocardial infarction; VT: venous thrombosis; VTE: venous thromboembolism; PE: pulmonary embolism; RA: rheumatoid arthritis; I/R injury, ischemia/reperfusion injury.

## Abbreviations

AA: Arachidonic acid
ACS: Acute coronary syndromes
AF: Atrial fibrillation
AP-1: Activator protein-1
APC: Activated protein C
aPL: Antiphospholipid antibody
APS: Antiphospholipid syndrome
AT: Arterial thrombosis
AT III: Antithrombin III
bFGF: Basic fibroblast growth factor
CHD: Coronary heart disease
COX: Cyclo-oxgyenase
CPB: Cardiopulmonary bypass
CRP: C-reactive protein
DIC: Disseminated intravascular coagulation
DVT: Deep vein thrombosis
EGF: Epidermal growth factor
Egr-1: Early growth reponse-1
FBG: Fibrinogen
FIIa: Thrombin
FVIIa: Activated factor VII
FVIIai: Active site inhibited FVIIa
FXa: Activated factor X
HMGCR: HMGCoA reductase
ICAM: Intracellular adhesion molecule
IL: Interleukin
LDL: Low-density lipoprotein
LMWH: Low-molecular-weight heparin
Lp(a): Lipoprotein (a)
LPS: Lipopolysaccharide; bacterial endotoxin
MAPK: Mitogenic activating protein kinase
MCP: Monocyte chemotactic protein
MI: Myocardial infarction
MMP: Matrix metalloproteinase
NF-*κ*B: Nuclear factor-kappa B
NO(S): Nitric oxide (synthase)
OxLDL: Oxidized LDL
PA: Plasminogen activator
PAF: Platelet activating factor
PAI-1: Plasminogen activator inhibitor-1
PAR: Protease activated receptor
PC: Protein C
PCA: Procoagulant activity
PDGF: Platelet derived growth factor
PE: Pulmonary embolism
PG: Glycoproteins
PGE2/J2: Prostagandin E2/J2
PKC: Protein kinase C
PPAR: Peroxisomal proliferation activated receptor
PS: Phosphatidylserine
RA: Rheumatoid arthritis
TAP: Tick anticoagulant peptide
TAT: Thrombin-antithrombin complex
TF: Tissue factor
TFPI: TF pathway inhibitor
TGF: Transforming growth factor
TLR: Toll-like receptors
TNF-*α:*: Tissue necrosis factor-alpha
VCAM: Vascular adhesion molecule
VEGF: Vascular endothelial growth factor
VT: Venous thrombosis
VTE: Venous thromboembolism.
